# Cryptorchids and Their Castration1Read at the June meeting of the Missouri Valley Veterinary Association.

**Published:** 1896-07

**Authors:** R. C. Moore

**Affiliations:** Kansas City, Mo.


THE JOURNAL
OF
COMPARATIVE MEDICINE AND
VETERINARY ARCHIVES.

VOL. XVII. *JULY*, 1896. No. 7.

### CRYPTORCHIDS AND THEIR CASTRATION.[1]

BY R. C. MOORE, V.S.,
KANSAS CITY, MO.

THE word cryptorchidism is applied to that condition in animal life where one or both of the testicles fail to descend into the scrotum. Its Latin roots signify "I conceal," and "a testicle," and might be translated a hidden testicle. In this country the words "ridgling" and "original" are frequently applied to animals presenting this condition. Webster defines the words ridgling and original, in respect to this subject, to mean a half-castrated animal, a sheep having only one testicle, a barrow hog, a half-castrated cock. So if we are to confine ourselves strictly to Webster's idea of the meaning of these words we should not apply them to the abnormal condition under discussion, as it is not necessary that an animal be half-castrated to have a hidden testicle. As custom largely governs the meaning of a word, regardless of Webster, we will not be considered greatly wrong if we apply the word ridgling to an animal having one or both testicles concealed, "not in the scrotum," for in this country it has been used to convey this idea for many years.

All male animals are subject to this abnormal condition, and I have found it to exist in the horse, ox, and hog, but so far have never met with it in the ass, mule, or dog. In this country but few asses are castrated, and the chances of finding this condition are greatly reduced. The mule is generally free from abnormal conditions, hardy, and tough, which may in a degree account for the seeming scarcity of cryptorchids among these hybrids.

[1] Read at the June meeting of the Missouri Valley Veterinary Association.

The infrequency of castration of the dog may account for my not having met with it in the canine race.

It is generally considered that cryptorchidism produces a marked unfavorable impression upon animals. In the horse, at least, this idea is certainly well founded in many cases. If there is a disagreeable, unpleasant, ill-tempered, devilish animal, it is certainly a mean ridgling horse. In other cases horses retain one or both testicles concealed until a good old age without developing a bad disposition, not worse than any entire horse. However, I believe this to be the exception, not the rule.

Cryptorchids may be divided into four classes: First, external inguinal; second, high or internal inguinal; third, abdominal; and fourth, infrascrotal.

The first is simply a short cord, drawing the testicle up to or into the external inguinal ring, and it is doubtful if they may be classed as ridglings, for the testicle is not hidden to a careful and experienced observer.

In the second class, the high, or internal inguinal, the testicle is hidden in the canal, or engaged within the internal inguinal or abdominal ring, or the testicle may lie on the inside of the abdomen with the epididymis or a loop of the spermatic cord extending down through the ring. In either of these conditions the tunica vaginalis, or vaginal sheath, is formed and occupying the inguinal canal, and is occupied by the testicles and cord, or so much of them as may have descended through the ring. If no part of the testicle or cord have entered the internal inguinal ring, then no vaginal sheath has been formed.

In the third class, abdominal cryptorchids, the testicle floats in the abdominal cavity, suspended by the testicular ligament, which is formed by a fold of peritoneum, dipping down from the antero-inferior face of the ileum, encircling the spermatic cord and vessels, and reflected over the epididymis and testicle. The parietal attachment of this fold extends from the margin of the internal or abdominal ring upward and forward for some distance and allows the testicle to float in the posterior part of the abdominal and anterior part of the pelvic cavities.

The fourth class, or infrascrotal, is one that so far I have failed to find described in veterinary literature, and yet one that is quite common in bovines. The testicle, after passing down through the inguinal ring and canal into the vaginal sheath, fails to press the dartos and skin down to form the scrotum. Instead of lodging here it passes forward along the course of the penis between the skin and abdominal tunic, sometimes reaching nearly to the end of the penis. This is doubtless due to the arrangement of the dartos, which is continuous with the superficial layer of the aponeurotic sheath of the penis of bovines (Chauveau's *Anatomy*, page 978).

It is sometimes important to decide whether or not a horse is a cryptorchid, and frequently it is a very difficult task. If a true history could always be obtained, it would be a very easy matter; but, alas! this is seldom the case and often impossible. The veterinarian is often left in doubt as to the truth. If we can be assured that the horse has never been castrated, and one or both testicles are missing from the scrotum, and their presence in the inguinal region cannot be detected by a careful inspection of that region, then we are safe in saying that the horse has a hidden testicle. If no history is obtainable, and the horse exhibits the usual signs of the stallion in appearance, build, action, and particularly in the voice, and no testicle is visible, we may reasonably conclude that he is a ridgling. Cicatrices on one or both sides of the scrotum is not evidence that the horse has been completely castrated, for many castrators attempt the operation and fail. Many geldings will tease a mare, and some will copulate; but the act is short and imperfect and easily detected as abnormal, and the voice will not have the coarse, husky tone of the stallion, while copulation by the ridgling will correspond in duration to that of the stallion. Again, we may find a horse that is undoubtedly a ridgling with positive evidence of having been cut into on both sides, and we are at a loss to know on which side the hidden testicle is lòcated. If the end of the spermatic cord cannot be found in either inguinal region, and there is no other evidence, I prefer the left side, as my own experience has led me to believe that much more than 50 per cent. of crpytorchids have the hidden testicle on the left side.

W. B. E. Miller, D.V.S. (*American Veterinary Review*, May, 1894, page 91), excludes all animals from the class of ridglings except those which have undeveloped testicles floating in the abdominal cavity. The word undeveloped will apply to the hidden testicle of most cryptorchids, but not to all, as I have on several occasions found as well-developed testicles floating in the abdominal cavity as I ever found in the scrotum. Experience has also led me to believe the more perfectly the hidden testicle is developed, no matter where it is located, the more the animal will resemble the normal male.

Where surgical interference in these cases has previously been attempted and failed, castration is rendered more difficult and the prognosis more unfavorable, because the mutilation and modification of the inguinal region may be very considerable, and the operation should not be undertaken without informing the owner of the increased risk. I believe it is a good rule never to shoulder the bad results of another's mistakes.

The castration of cryptorchids is much more difficult than the normal horse, and was considered very dangerous, but at the present time it is not more so than other serious and difficult operations. In ordinary cases and under reasonable surgical precautions, provided patients have good after-treatment, the mortality should not exceed 3 per cent. The preparation of the horse for the operation and his subsequent treatment have largely to do with the prognosis. The horse should be in good general health, bowels loose from previous soft foods, and the alimentary canal should be as free from food as possible. Abstinence from everything except bran-mashes for forty-eight hours is advisable. A full abdomen makes the operation difficult to perform, and the prognosis doubtful. The instruments required are a castrating-knife and écraseur or emasculator. The surgical procedure is rendered easier by selecting a hillside for the operation. By placing the animal's back and head down the hill it is easier to keep him on his back, and, by the head being the lowest, the abdominal viscera will gravitate forward and lessen the tension of the posterior part of the abdominal wall, which greatly facilitates manipulation. The horse should be cast and tied as securely as possible, with the hocks and stifles drawn as far back as convenient, and the thighs spread well apart, so as to expose the inguinal regions. An anæsthetic may be used if preferred, but it is desirable to have the horse regain his feet after the operation in as steady a manner as possible, and this is not always obtainable when an anæsthetic has been used.

The horse being in proper position, incision is made in the scrotal region, commencing four inches back of the point of the sheath, and one or two inches from the median line, and carried backward parallel with the median line about four inches, or sufficient to admit the hand, cutting through the skin and dartos to the external inguinal ring. The cellular tissue here may be separated with the fingers, the hand anointed with carbolized oil, and the fingers formed into a cone. By a rotary motion a canal can be opened up to the normal location of the internal inguinal ring, or the lower margin of an imperfectly developed canal. If the ring cannot be found, its location may be determined by measuring about three inches from the insertion of prepubic tendon to the symphysis pubis, along the course of Poupart's ligament. If the testicle or a loop of the cord has passed through, it will be found in the inguinal canal enclosed in the vaginal sheath. If found, it should be grasped firmly and brought to the surface, and a free incision made in the vaginal sheath; the cord should then be severed as in a straight colt.

In operating on this class the most important point is the incision of the vaginal sheath. It requires no little force to bring a short vaginal sheath to the surface, and the pressure thus applied to the testicle will cause it to push through a very small incision in the sheath. When the cord is severed the stump will return within the sheath. Infiltration and tumefaction soon close the small opening in the sheath and prevent the escape of the accumulations from above—a serious complication. To obviate this it is only necessary to make a free opening in the vaginal sheath. If I fail to accomplish this before the testicle escapes from the sheath, I enlarge the incision with a probe bistoury before severing the cord.

If the testicle or cord is not found in the canal it is an abdominal ridgling, and the operation becomes more difficult. In this case the end of the finger should be passed upward and outward somewhat beyond the margin of the internal inguinal ring, keeping back close to Poupart's ligament, and by a gentle pressure of the finger break down the peritoneal wall. This will bring the finger into the peritoneal sac at the attachment of the testicular ligament. The ligament or serous fold should then be grasped with two fingers and brought to the outside, and the testicle will follow. The operation is accomplished by severing the cord by the écraseur or emasculator.

It is not impossible for even an experienced surgeon to fail to properly locate the abdominal ring and get too far forward (Poupart's ligament will prevent one from going too far back). In this case it will be difficult to find the testicular ligament, and it may be necessary to insert the hand and search for the testicle. A large opening in the abdominal wall so far forward would be very liable to be followed by hernia of the small intestine, or a loop of intestine may be mistaken for the spermatic cord and pulled down. While this is a serious complication, it is not necessarily fatal. Should this occur after applying the écraseur to the cord, the intestines should be returned, and the artificial inguinal canal packed with antiseptic gauze, or a soft cotton cloth, if other material is not at hand, then sever the cord. If the intestines are difficult to return the operation may be facilitated by passing the hand into the rectum and over the inguinal region, holding the intestines back as they are replaced by the other hand. A stitch or two may be taken if the peritoneal opening is large, and the horse allowed to arise at once, turning him over that he may arise from the side from which the testicle has just been removed. This cramps the inguinal region and reduces the danger of hernia or its recurrence.

It is necessary to use some care not to wound the blood-vessels in the inguinal region. The internal pudic veins form a very rich network above the penis, between the thighs, and in the texture of the scrotum. They are large and anastomose freely with each other, and are liable to be wounded in opening the external inguinal ring and inguinal canal. If this accident does occur it is generally necessary to ligate both ends of the divided vessels, as the pressure from the cramped position of the horse while cast will often prevent them from bleeding, but as soon as he is on his feet they will bleed profusely. The prepubic artery, after crossing the crural ring, gains the posterior aspect of the internal inguinal ring, and divides into the posterior abdominal and external pudic, the latter following down the canal to the external inguinal ring. Either of these may be wounded. To avoid this it is only necessary to remember that they lay internal to the vaginal sheath and internal inguinal ring, and all incisions should be made external to these parts.

The after-treatment will, to a greater or less degree, depend upon the surroundings. If the surgeon can have the patient under his own care, with sufficient sanitary conditions to warrant thorough antisepsis, then healing by first intention may be expected. To accomplish this it will be imperative to use the utmost precaution before, during, and after the operation. Operating-room, tables, sponges, and all instruments should be clean and sterilized. It is best to boil all instruments and sponges, scald tables, floors, and everything that cannot be boiled. If the patient is secured on the floor, clean straw or sawdust for bedding may be used, which should be thoroughly sprinkled, preferably with an antiseptic solution, to prevent the escape of all dust from the bedding. When the horse is cast and properly secured, the inguinal regions should be thoroughly cleansed with soap and water. Before operating those parts should be thoroughly disinfected with the bichloride solution, or any other good disinfectant. The surgeon's hands should be thoroughly cleansed and disinfected. All instruments, after being thoroughly boiled, should be placed on a clean tray, covered with a towel saturated with the disinfectant solution. After the operation the wound should be thoroughly cleansed and dried and sutured, then a dry antiseptic dressing applied. When the animal has regained his feet a pad of absorbent cotton dusted with iodoform or some other dry dressing should be applied to the wound and secured with bandages over the loins and between the thighs, which should be renewed as required.

If, as is usually the case in country districts, the patient cannot have the best of antiseptic treatment, then healing by first intention does not seem to be feasible. Even then the surgeon should use every reasonable antiseptic precaution, but instead of suturing the wound it should be left open, and carbolized oil applied, which will tend to keep the wounded surfaces from adhering. The wound must then be opened once a day by inserting the fingers, well oiled, up to the internal inguinal ring, or the wound may be packed with antiseptic gauze, this to be removed twenty-four to thirty-six hours later. This insures good drainage and prevents integumentary adhesions. The patient should be tied, so that he cannot lie down. Colicky pains are sometimes present, and the horse is liable to roll, and if allowed to do so hernia may be produced. After the first day he should have plenty of exercise. Do not depend upon him taking enough exercise in a paddock or small yard, for but few will do it. One hour's steady walk is better than all the exercise he would take in twenty-four hours alone. If he is a work-horse, he can do his usual work every day after the first day. If peritonitis or other complications set in, they must be given appropriate treatment.

If the horse proves to be a double ridgling, both testicles may be removed at one time, if the surgeon prefers; or the patient may be allowed to recover from the wound by the removal of one before taking the other. If the first testicle is secured without much injury to the parts and without serious complications, I prefer to remove the second one at once; but in case of a difficult or complicated operation it is good practice to wait for recovery.

In the ridgling bull is usually found the infrascrotal form, and by feeling along the course of the penis the testicle is readily located. It may be removed where so found, the same as if in the scrotum. If it proves to be in the abdomen, it can be removed through the flanks, as in spaying. This is also the best method in the pig.

